# miR-1269a and miR-1269b: Emerging Carcinogenic Genes of the miR-1269 Family

**DOI:** 10.3389/fcell.2022.809132

**Published:** 2022-02-18

**Authors:** Zijun Xie, Chenming Zhong, Shiwei Duan

**Affiliations:** ^1^ School of Medicine, Zhejiang University City College, Hangzhou, China; ^2^ Medical Genetics Center, School of Medicine, Ningbo University, Ningbo, China; ^3^ Department of Clinical Medicine, Zhejiang University City College School of Medicine, Hangzhou, China

**Keywords:** miR-1269a, miR-1269b, target gene, non-coding RNAs, prognosis

## Abstract

miRNAs play an important role in the occurrence and development of human cancer. Among them, hsa-mir-1269a and hsa-mir-1269b are located on human chromosomes 4 and 17, respectively, and their mature miRNAs (miR-1269a and miR-1269b) have the same sequence. miR-1269a is overexpressed in 9 cancers. The high expression of miR-1269a not only has diagnostic significance in hepatocellular carcinoma and non-small cell lung cancer but also is related to the poor prognosis of cancer patients such as esophageal cancer, hepatocellular carcinoma, and glioma. miR-1269a can target 8 downstream genes (CXCL9, SOX6, FOXO1, ATRX, RASSF9, SMAD7, HOXD10, and VASH1). The expression of miR-1269a is regulated by three non-coding RNAs (RP11-1094M14.8, LINC00261, and circASS1). miR-1269a participates in the regulation of the TGF-β signaling pathway, PI3K/AKT signaling pathway, p53 signaling pathway, and caspase-9-mediated apoptotic pathway, thereby affecting the occurrence and development of cancer. There are fewer studies on miR-1269b compared to miR-1269a. miR-1269b is highly expressed in hepatocellular carcinoma, non-small cell lung cancer, oral squamous cell carcinoma, and pharyngeal squamous cell carcinoma, but miR-1269b is low expressed in gastric cancer. miR-1269b can target downstream genes (METTL3, CDC40, SVEP1, and PTEN) and regulate the PI3K/AKT signaling pathway. In addition, sequence mutations on miR-1269a and miR-1269b can affect their regulation of cancer. The current studies have shown that miR-1269a and miR-1269b have the potential to be diagnostic and prognostic markers for cancer. Future research on miR-1269a and miR-1269b can focus on elucidating more of their upstream and downstream genes and exploring the clinical application value of miR-1269a and miR-1269b.At present, there is no systematic summary of the research on miR-1269a and miR-1269b. This paper aims to comprehensively analyze the abnormal expression, diagnostic and prognostic value, and molecular regulatory pathways of miR-1269a and miR-1269b in multiple cancers. The overview in our work can provide useful clues and directions for future related research.

## Introduction

MicroRNA (miRNA) is an endogenous non-coding RNA with a length of 20–22 nucleotides, which can usually bind to the 3′-untranslated region of its target gene to silence gene expression (Ambros, 2004). hsa-mir-1269a at chromosome 4 and hsa-mir-1269b at chromosome 17 are members of the miR-1269 family. They can produce mature miRNAs (miR-1269a and miR-1269b) (Kong et al., 2016) with the same sequence.

At present, there are many bioinformatics studies on miRNA. Tens of miRNA research tools are integrated on the tools4mirs website (https://tools4mirs.org/) (Lukasik et al., 2016). miRNA target gene prediction tools mainly used in the miR-1269 related studies include TargetScan (http://www.targetscan.org/) (McGeary et al., 2019), miRDB (http://www.mirdb.org/miRDB/) (Chen and Wang, 2020), and mirwalk (http:///mirwalk.umm.uni-heidelberg.de/) (Sticht et al., 2018).

miR-1269a is abnormally highly expressed in 9 cancers, used for the diagnosis of 6 cancers, and is also related to the prognosis of 6 cancers. miR-1269a is also involved in the occurrence and progression of diseases other than cancer. For example, the high expression of miR-1269a may be a risk factor for ectopic pregnancy (Zhang et al., 2018). miR-1269a can regulate the expression of 8 downstream genes and is related to the regulation of three signaling pathways. As to miR-1269b, it is abnormally expressed in 4 kinds of cancers (3 kinds of high expression, one kind of low expression), and is related to the prognosis of two kinds of cancers. miR-1269b can regulate 4 downstream genes and participate in two signaling pathways. In addition, the genetic variants of both miR-1269a and miR-1269b can affect the function of their wild types.

Although there are many reports on miR-1269a and miR-1269b, there is no systematic summary of the two miRNAs. Because miR-1269a and miR-1269b have the same sequence and similar names, researchers may confuse these two miRNAs. Therefore, this article summarizes the abnormal expression of miR-1269a and miR-1269b in various cancers and their diagnostic and prognostic value in cancer. In addition, this article comprehensively analyzes the molecular regulation pathways related to miR-1269a and miR-1269b, which is expected to provide guidance for future related research.

## Oncological Role of miR-1269a and miR-1269b in Cancer

miR-1269a is highly expressed in 9 cancers, including hepatocellular carcinoma (Wojcicka et al., 2014; Yang et al., 2014; Gan et al., 2015; Elemeery et al., 2017; Wang et al., 2019; Cho et al., 2020), lung cancer (Bao et al., 2018; Jin et al., 2018; Guo et al., 2020; Wang et al., 2020; Du et al., 2021; Le and Le, 2021), gastric cancer (Liu et al., 2019; Zhang K. et al., 2020), colon cancer (Bu et al., 2015; Xiong et al., 2021), esophageal cancer (Bai et al., 2021; Yu and Ren, 2021; Zhao et al., 2021), clear cell renal cell carcinoma (Qin et al., 2019; Zhan et al., 2021), head and neck squamous cell carcinoma (Nunez Lopez et al., 2018), glioma (Zhang Y. et al., 2020), and acute myeloid leukemia (Li and Ge, 2021). Highly expressed miR-1269a can promote cancer cell proliferation, migration, invasion, epithelial-mesenchymal transition, and inhibit cancer cell apoptosis (Table 1). It is worth noting that in hepatocellular carcinoma (Xiong et al., 2015; Min et al., 2017) and gastric cancer (Li et al., 2017), there are highly expressed miR-1269a mutant and low expressed miR-1269a wild type. Interestingly, the miR-1269a mutant can inhibit the cancer-promoting effect of wild-type miR-1269a, which provides very valuable evidence for targeted cancer therapy.

**TABLE 1:** The role of miR-1269a and miR-1269b in different human cancers.

miR-1269a/b	Tumor type	Expression pattern	Number of clinical samples	Assessed cell lines	Effect *in vitro*	Effect *in vivo*	Regulatory mechanism	Ref
miR-1269a								
	HCC	Up-regulated	723 cases and 698 controls	HepG2 and SMMC-7721	Proliferation↑, Apoptosis ↓	—	miR-1269a/SPATS2L and LRP6 axis	Min et al. (2017)
	HCC	Up-regulated	590 cases and 549 controls	HepG2 and Huh7	Proliferation↑	—	miR-1269a/SOX6 axis	Xiong et al. (2015)
	HCC	Up-regulated	23 paired tissues	HepG2, Huh7, Hep3B, THLE3, BEL-7402, BEL-7404, SNU-398, SNU-449, and QGY-7703	Proliferation↑, Tumorigenicity↑, Cell cycle↑	—	miR-1269a/FOXO1 axis	Yang et al. (2014)
	HCC	Up-regulated	24 paired tissues	—	—	—	—	Wojcicka et al. (2014)
	HCC	Up-regulated	375 cases and 50 controls	—	—	—	—	Wang et al. (2019)
	HCC	Up-regulated	108 tissues and 720 serums	—	—	—	—	Cho et al. (2020)
	HCC	Up-regulated	95 paired tissues	—	—	—	—	Gan et al. (2015)
	HCC	Up-regulated	474 cases and 84 controls	—	—	—	—	Elemeery et al. (2017)
	GC	Up-regulated	373 paired tissues and 402 controls	MGC803 and HGC27	Proliferation↑, Apoptosis↓	—	miR-1269a/ZNF70 axis	Li et al. (2017)
	GC	Up-regulated	—	AGS, MKN45, NCI-N87, MGC803, and GES-1	—	Tumor growth↑	RP11-1094M14.8/miR-1269a/CXCL9 axis	Zhang K. et al. (2020)
	GC	Up-regulated	73 paired tissues	AGS, MKN45, BGC-823, SGC7901, and GES-1	Proliferation↑, Cell cycle↑, Apoptosis↓	—	miR-1269a/RASSF9 axis	Liu et al. (2019)
	NSCLC	Up-regulated	147 peripheral blood samples and 149 controls	A549 and H1975	Proliferation↑, Migration and Invasion↑, EMT↑	—	miR-1269a/FOXO1 axis	Wang et al. (2020)
	NSCLC	Up-regulated	49 paired tissues	A549, SPC-A1, PC-9, H1299, H1975, H460, and BEAS-2B	Proliferation↑, Colony formation↑, Cell cycle↑	—	miR-1269a/SOX6 axis	Jin et al. (2018)
	NSCLC	Up-regulated	134 cases and 50 controls	—	—	—	—	Le and Le, (2021)
	LC	Up-regulated	78 paired tissues	A549, SPC-A1, CBP60577, NCI-H1299, NCI-H23, L78, and BEAS-2B	Proliferation↑, Migration and Invasion↑, Cell cycle↑, Apoptosis↓	Tumor growth↑	LINC00261/miR-1269a/FOXO1 axis	Guo et al. (2020)
	LC	Up-regulated	52 paired tissues	A549	Proliferation↑, Apoptosis↓	—	miR-1269a/TP53 and CASP9 axis	Bao et al. (2018)
	CRC	Up-regulated	100 cases	HCT116, LoVo, HT29, SW480, SW620, DLD1, and LS174T	Migration and Invasion↑, EMT↑	Tumor growth and metastasis↑	TGF-β positive feedback pathway	Bu et al. (2015)
	CRC	Up-regulated	10 paired tissues	HCT116, LoVo, HT29, SW480, Caco2, and HIEC 6	Proliferation↑, Migration and Invasion↑, Apoptosis↓	—	circASS1/miR-1269a/VASH1 axis	Xiong et al. (2021)
	ESCC	Up-regulated	107 paired tissues	Eca-109, TE-1, KYSE-150, TE-10, and Het-1A	Proliferation↑, Migration and Invasion↑	—	miR-1269a/SOX6 axis	Bai et al. (2021)
	ccRCC	Up-regulated	480 cases and 68 controls	—	—	—	—	Qin et al. (2019)
	Glioma	Up-regulated	107 paired tissues; 84 cases and 10 controls	U251, SNB19, SHG44, A172, and HEB	Progression↑, Migration and Invasion↑, Apoptosis ↓	Tumor growth↑	miR-1269a/ATRX axis	Zhang Y. et al. (2020)
	PC	Up-regulated	135 cases	PC3, DU145, LNCaP, 22Rv1, VCaP, and HT-1080	—	—	—	Scaravilli et al. (2015)
	AML	Up-regulated	47 cases and 32 controls	—	—	—	—	Li and Ge, (2021)
miR-1269b								
	HCC	Up-regulated	—	HepG2, SMMC-7721, and HepG2.2.15	Proliferation↑, Migration↑, Cell cycle↑	—	HBx/NF-κB/miR-1269b/CDC40 axis	Kong et al. (2016)
	HCC	Up-regulated	220 cases	Huh7, Hep3B, PLC, HLE, MHCCLM3, MHCC97H, and MHCC97L	Proliferation↑, Migration and Invasion↑, Chemotaxis↑	Tumor growth and metastasis↑	miR-1269b/SVEP1; PI3K/AKT pathways	Chen et al. (2020)
	HCC	Up-regulated	415 cases and 334 controls	—	—	—	—	Ma et al. (2020)
	GC	Down-regulated	143 paired tissues	AGS, NCI-N87, HGC27, SNU-16, and GES-1	Proliferation↓, Migration and Invasion↓	—	miR-1269b/METTL3 axis	Kang et al. (2021)
	NSCLC	Up-regulated	32 paired tissues	A549, A549/DDP, SPC-A1, PC-9, H1299, H358, and 16HBE	Proliferation↑, Apoptosis↓, Drug resistance↑	Tumor growth↑	miR-1269b/PTEN; PI3K/AKT pathways	Yang et al. (2020)
	OPSCC	Up-regulated	1087 cases and 865 controls	—	—	—	—	Chen et al. (2016)

HCC, hepatocellular carcinoma; GC, gastric cancer; NSCLC, Non-small cell lung cancer; LC, lung cancer; CRC, colorectal cancer; ESCC, esophageal squamous cell carcinoma; ccRCC, clear cell renal cell carcinoma; PC, prostate cancer; AML, acute myeloid leukemia; OPSCC, oropharyngeal squamous cell carcinoma; ↑, Promotion; ↓, Inhibition.

Similarly, miR-1269b is also highly expressed in hepatocellular carcinoma (Kong et al., 2016; Chen et al., 2020; Ma et al., 2020), lung cancer (Yang et al., 2020), and oral and pharyngeal squamous cell carcinoma (Chen et al., 2016). Overexpression of miR-1269b can down-regulate METTL3, thereby inhibiting the proliferation, migration, and invasion of gastric cancer cells (Kang et al., 2021). It is worth noting that miR-1269b is low expressed in gastric cancer (Table 1).

Changes in miRNA expression are a fundamental component of cancer progression. The current study shows that the aberrant expression of miR-1269a or miR-1269b is present in a variety of cancers. Overall, abnormal expression of miR-1269a and miR-1269b can promote or interfere with the occurrence and development of cancer by regulating biological processes such as cancer cell proliferation, migration, invasion, apoptosis, and epithelial-mesenchymal transition. The abnormal expression of miR-1269a and miR-1269b may reflect the regulation of ceRNAs or other upstream genes in different tumors. We also observed that different detection methods of miR-1269a/b were used in these studies. As shown in Supplementary Table S1, qRT-PCR is commonly used to detect the expression of miR-1269a (Yang et al., 2014; Bu et al., 2015; Gan et al., 2015; Scaravilli et al., 2015; Kong et al., 2016; Li et al., 2017; Min et al., 2017; Jin et al., 2018; Liu et al., 2019; Zhang K. et al., 2020; Zhang Y. et al., 2020; Chen et al., 2020; Cho et al., 2020; Guo et al., 2020; Bai et al., 2021; Kang et al., 2021; Li and Ge, 2021; Xiong et al., 2021). Some studies have also applied RT-PCR technology to detect miR-1269 expression for hepatocellular carcinoma (Xiong et al., 2015; Elemeery et al., 2017), lung cancer (Elemeery et al., 2017; Wang et al., 2020), and oropharyngeal squamous cell carcinoma (Chen et al., 2016). In addition, studies in hepatocellular carcinoma and acute myeloid leukemia used next-generation sequencing technology (Wojcicka et al., 2014; Chen et al., 2020; Cho et al., 2020) and transcriptome high-throughput sequencing (Li and Ge, 2021) to detect miR-1269. Most of the studies provided the primer sequences used in the experiments, but some studies directly used the data in the database without providing the corresponding primer sequences (Wojcicka et al., 2014; Yang et al., 2014; Bu et al., 2015; Scaravilli et al., 2015; Xiong et al., 2015; Chen et al., 2016; Elemeery et al., 2017; Min et al., 2017; Bao et al., 2018; Yang et al., 2020; Bai et al., 2021; Le and Le, 2021; Li and Ge, 2021). Furthermore, we notice that there is a microarray platform (Illumina HumanHT-12 V4.0 expression beadchip) that can detect miR-1269a, but not miR-1269b. Therefore, the current few studies of miR-1269b may be related to relatively few detection methods.

## The Diagnostic and Prognostic Value of miR-1269

As shown in Table 2, the high expression of miR-1269a is closely related to the clinicopathological characteristics of cancer patients. In hepatocellular carcinoma, high expression of miR-1269a is significantly positively correlated with vascular invasion and TNM staging (Gan et al., 2015). In lung cancer, highly expressed miR-1269a is significantly associated with lymph node metastasis and advanced TNM staging (Guo et al., 2020; Wang et al., 2020; Le and Le, 2021). In addition, in esophageal squamous cell carcinoma, highly expressed miR-1269a is significantly associated with poor tumor differentiation, lymph node metastasis, and TNM staging (Jang et al., 2017; Bai et al., 2021; Yu and Ren, 2021).

**TABLE 2:** The prognostic value of miR-1269a and miR-1269b in cancers.

miR-1269a/b	Tumor type	Sample size	Expression pattern	Prognostic/Diagnostic value	Ref
miR-1269a					
	HCC	254 patients	Up-regulated	Prognostic factor of OS and DFS	Cho et al. (2020)
	HCC	95 patients	Up-regulated	Positively associated with vaso-invasion, multiple tumor nodes and TNM stage; AUC = 0.640	Gan et al. (2015)
	HCC	224 patients	Up-regulated	Positively associated with late fibrosis; AUC = 0.691, sensitivity = 0.786, specificity = 0.598	Elemeery et al. (2017)
	ESCC	322 patients	Up-regulated	Positively associated with TNM stage; prognostic factor of OS and RFS	Jang et al. (2017)
	ESCC	107 patients	Up-regulated	Positively associated with lymph node metastasis and TNM stage; Prognostic factor of OS	Bai et al. (2021)
	ESCC	125 patients	Up-regulated	Positively associated with low differentiation, lymph node metastasis, TNM stage and AJCC stage; Prognostic factor of OS (AUC = 0.716) and CSS (AUC = 0.764)	Yu and Ren, (2021)
	NSCLC	147 patients	Up-regulated	AUC = 0.793	Wang et al. (2020)
	NSCLC	84 patients	Up-regulated	Positively associated with lymph node metastasis and TNM stage; AUC = 0.906, sensitivity = 0.86, specificity = 0.833	Le and Le, (2021)
	LC	78 patients	Up-regulated	Positively associated with lymph node metastasis and TNM stage; Prognostic factor of OS	Guo et al. (2020)
	ccRCC	480 patients	Up-regulated	Prognostic factor of OS	Qin et al. (2019)
	ccRCC	512 patients	Up-regulated	Prognostic factor of OS	Zhan et al. (2021)
	Glioma	99 patients	Up-regulated	Prognostic factor of OS	Zhang Y. et al. (2020)
miR-1269b					
	HCC	—	Up-regulated	Prognostic factor of OS	Ma et al. (2020)
	NSCLC	32 patients	Up-regulated	Prognostic factor of OS	Yang et al. (2020)

HCC, hepatocellular carcinoma; ESCC, esophageal squamous cell carcinomas; NSCLC, Non-small cell lung cancer; LC, lung cancer; ccRCC, clear cell renal cell carcinoma; OS, overall survival; DFS, Disease-free survival; TNM, Tumour-node-metastasis; AUC, area under the curve; RFS, Recurrence-free survival; AJCC, american joint committee on cancer; CSS, Cancer-specific survival.

ROC analysis showed that the AUC of miR-1269a expression was 0.640, indicating that the level of miR-1269a has a certain diagnostic value for hepatocellular carcinoma (Gan et al., 2015). In addition, the sensitivity, specificity, and AUC for miR-1269a were 0.598, 0.786, and 0.691 in the classification between liver fibrosis patients and hepatocellular carcinoma patients, indicating that miR-1269a can be used as a biomarker to track the progression of liver fibrosis to hepatocellular carcinoma (Elemeery et al., 2017). ROC curve analysis showed that the sensitivity, specificity, and AUC for miR-1269a in the diagnosis of lung cancer were 0.833, 0.86, and 0.906 (Le and Le, 2021). In addition, miR-1269a in serum exosomes might be used to diagnose tumors. The current study showed that serum exosomal miR-1269a can be used as a diagnostic marker for hepatocellular carcinoma (Cho et al., 2020) and non-small cell lung cancer (Wang et al., 2020).

The expression levels of miR-1269a and miR-1269b are significantly related to the prognosis of cancer patients (Table 2). The high expression of miR-1269a is significantly associated with the lower overall survival (OS) of 6 kinds of cancer patients, including hepatocellular carcinoma (Cho et al., 2020), esophageal squamous cell carcinoma (Jang et al., 2017; Bai et al., 2021; Yu and Ren, 2021), lung cancer (Guo et al., 2020), clear cell renal cell carcinoma (Qin et al., 2019; Zhan et al., 2021), glioma (Zhang Y. et al., 2020) and acute myeloid leukemia (Li and Ge, 2021). In hepatocellular carcinoma, high expression of miR-1269a is significantly associated with shorter disease-free survival (DFS) in patients with hepatocellular carcinoma (Cho et al., 2020) and lower cancer-specific survival (CSS) in patients with esophageal squamous cell carcinoma (Yu and Ren, 2021). These results suggest that miR-1269a is expected to be a biomarker for predicting poor prognosis in cancer patients. Similarly, high expression of miR-1269b was significantly associated with lower overall survival in patients with hepatocellular carcinoma (Ma et al., 2020) and non-small cell lung cancer (Yang et al., 2020). Highly expressed miR-1269b is also associated with cisplatin resistance in patients with non-small cell lung cancer (Yang et al., 2020).

## Molecular Mechanism of miR-1269a in Tumor

### miR-1269a and its ceRNA Network

Competitive endogenous RNA (ceRNA) can link the function of protein-coding mRNA with the function of non-coding RNA (such as microRNA, long non-coding RNA, and circular RNA) (Qi et al., 2015). The ceRNAs of miR-1269a includes lncRNA RP11-1094M14.8, LINC00261, and circASS1, which can form the RP11-1094M14.8/miR-1269a/CXCL9 axis, LINC00261/miR-1269a/FOXO1 axis and circASS1/miR-1269a/VASH1 axis.

**FIGURE 1:**
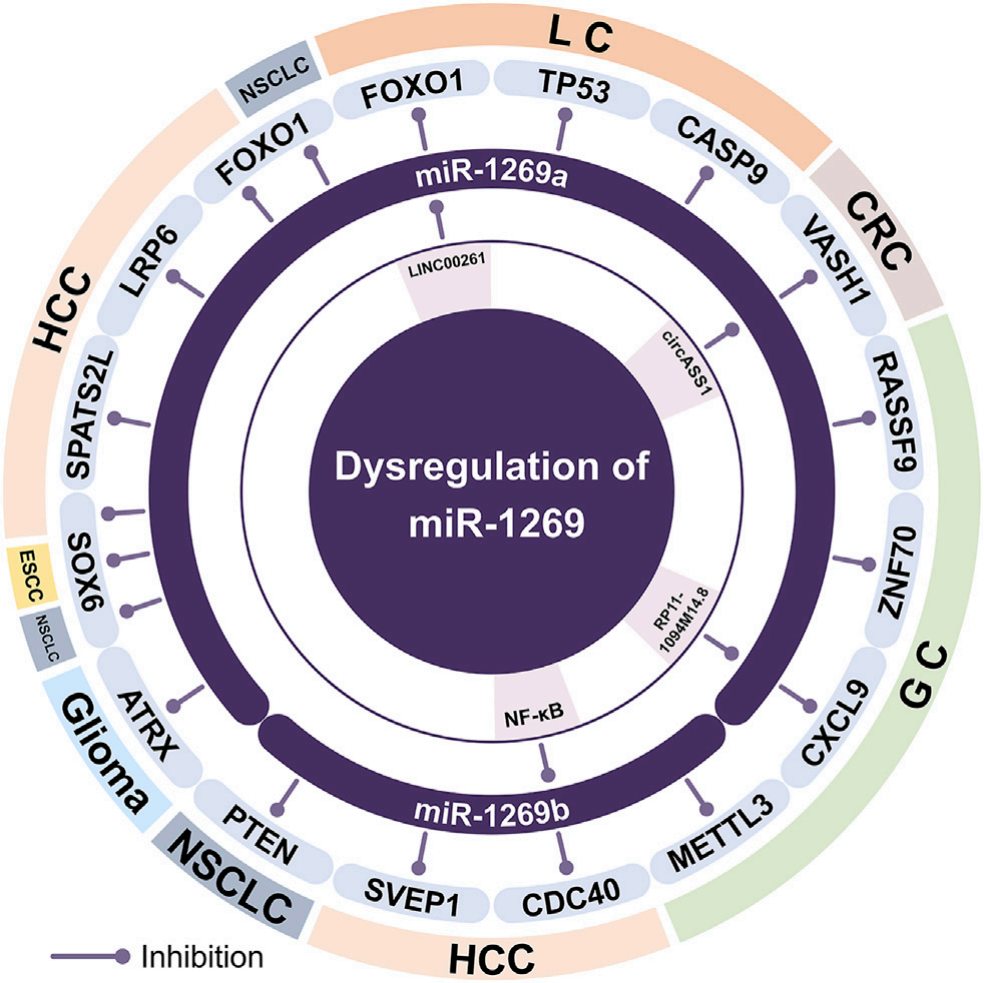
The dysregulation of miR-1269a and miR-1269b in different cancers. miR-1269a can be regulated by three upstream ncRNAs. miR-1269a and miR-1269b can regulate the occurrence and development of cancer by targeting downstream genes. HCC, Hepatocellular carcinoma; GC, Gastric cancer; CRC, Colorectal cancer; NSCLC, Non-small cell lung cancer; ESCC, Esophageal squamous cell carcinomas; LC, Lung cancer.

CXCL9 plays an important regulatory role in immune infiltration, and its expression level is significantly positively correlated with the infiltration of various immune cells such as NK cells, B cells, and dendritic cells (DCs) (Zhang K. et al., 2020). There is a lncRNA RP11-1094M14.8/miR-1269a/CXCL9 axis in gastric cancer. In gastric cancer specimens of immunotherapy patients, lncRNA RP11-1094M14.8 up-regulated the expression of CXCL9 by inhibiting miR-1269a, thereby promoting CXCL9-mediated lymphocyte infiltration into the lesion and inhibiting tumor growth (Zhang K. et al., 2020) (Figure 1).

FOXO1 is a key regulatory factor in the development of multiple organs or tissue cells. The absence of FOXO1 is more likely to promote the occurrence and development of tumors (Guo et al., 2020). The expression of LINC00261 is down-regulated in lung cancer, and the overexpression of LINC00261 inhibits the growth and metastasis of lung cancer by regulating the miR-1269a/FOXO1 axis (Guo et al., 2020) (Figure 1).

VASH1 is an endogenous angiogenesis inhibitor induced by VEGF and FGF-2 (Sato, 2013). VASH1 overexpression promotes cancer cell apoptosis and senescence and inhibits tumor occurrence and metastasis (Xiong et al., 2021). In colon cancer cells, the expression of circASS1 and VASH1 is reduced, and the high expression of circASS1 can down-regulate miR-1269a, thereby up-regulating VASH1 to inhibit the growth and metastasis of colon cancer (Xiong et al., 2021) (Figure 1).

### Downstream Genes of miR-1269a and Their Functions

miR-1269a can directly target SOX6, FOXO1, and ATRX to affect the occurrence and development of tumors (Figure 1). SOX6 is a member of the SOX transcription factor family, which is low expressed in a variety of tumors (Jin et al., 2018). SOX6 reduces tumor cell proliferation by promoting the expression of P21 and inhibiting CyClin D1 (Jin et al., 2018). In non-small cell lung cancer, miR-1269a can down-regulate SOX6 to promote tumor growth (Jin et al., 2018). In addition, in hepatocyte carcinoma (Xiong et al., 2015) and esophageal squamous cell carcinoma (Bai et al., 2021), the miR-1269a/SOX6 axis promotes cell proliferation, migration, and invasion, thereby promoting the development of tumors. FOXO1 is a transcriptional activation factor, which can regulate the expression of cell cycle blocking, apoptosis, DNA repair, and hypoxia reaction (Yang et al., 2014). In non-small cell lung cancer, miR-1269a can inhibit FOXO1 to promote cell proliferation, migration, and invasion (Wang et al., 2020). In hepatocellular carcinoma, miR-1269a/FOXO1 can up-regulate Cyclin D1, thereby promoting tumor cell proliferation (Yang et al., 2014). ATRX plays a vital role in chromatin remodeling and maintaining genome and telomere stability. It is one of the key molecular biomarkers for the classification and diagnosis of glioma (Zhang Y. et al., 2020). In glioma cells, the significantly increased expression of miR-1269a can promote the proliferation and invasion of glioma cells and inhibit apoptosis. miR-1269a can significantly down-regulate the expression of ATRX *in vivo* and *in vitro*, and the overexpression of ATRX can also reverse the tumor-promoting effect induced by miR-1269a (Zhang Y. et al., 2020).

### The Positive Feedback Regulation Between miR-1269a and TGF-β

**FIGURE 2:**
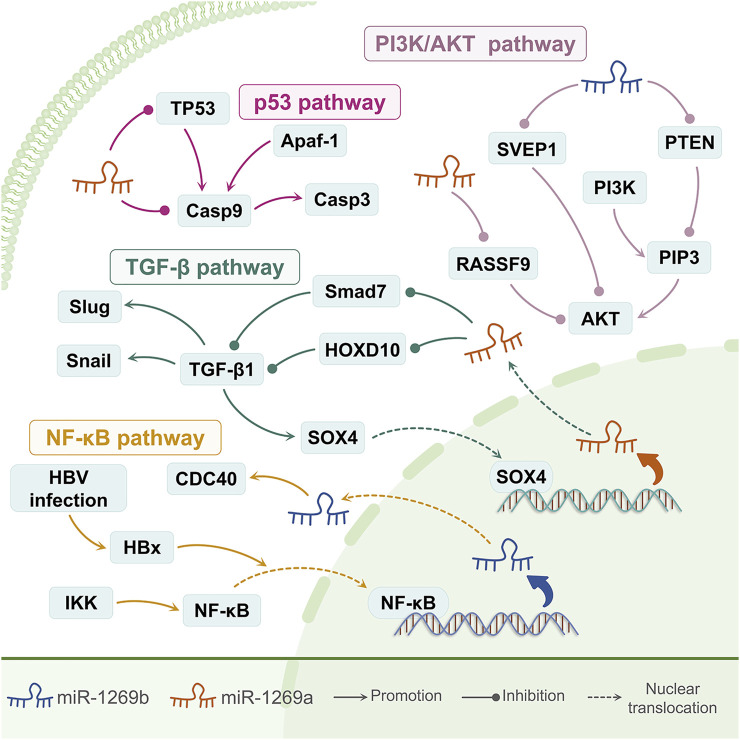
The signaling pathways involved with miR-1269a and miR-1269b. A schematic illustration of the roles of miR-1269a and miR-1269b in multiple signaling pathways. miR-1269a can inhibit apoptosis by inhibiting TP53, the core of the p53 signaling pathway, and Casp9, the initiation factor of apoptosis. miR-1269a up-regulates TGF-β signaling by suppressing its antagonists Smad7 and HOXD10. TGF-β, in turn, up-regulates miR-1269a *via* SOX4 to form a positive feedback loop in the TGF-β signaling pathway. miR-1269a also activates the PI3K/AKT signaling pathway by inhibiting RASSF9. miR-1269b participates in the activation of the PI3K/AKT signaling pathway by inhibiting SVEP1 and PTEN, and HBx promotes tumor progression by inducing miR-1269b to up-regulate CDC40 in an NF-κB dependent manner.

Transforming growth factor-β (TGF-β) family members play a vital role in cellular processes such as immunosuppression, growth inhibition, EMT, and cell invasion (Xie et al., 2018). In the late stages of cancer progression, the TGF-β signaling pathway can increase the expression of mesenchymal markers and reduce the expression of epithelial markers to promote EMT (Xie et al., 2018). In colorectal cancer, TGF-β can activate miR-1269a by promoting Sox4, inhibit SMAD7 and HOXD10, thereby enhancing TGF-β signaling and forming a positive feedback loop, promoting the EMT and metastasis of tumor cells (Bu et al., 2015) (Figure 2).

### miR-1269a and the p53 Signaling Pathway

p53 is an important tumor suppressor gene, and abnormalities of the p53 signaling pathway usually occur in tumors with higher malignancy (Bao et al., 2018). Caspase-9 is the initiation factor of cell apoptosis, and p53 can activate the caspase-9-mediated apoptotic pathway (Kim et al., 2015). In lung cancer, miR-1269a promotes lung cancer cell proliferation and inhibits apoptosis through targeted inhibition of p53 and caspase-9 (Bao et al., 2018) (Figure 1).

### miR-1269a and the PI3K/AKT Signaling Pathway

The PI3K/AKT signaling pathway can regulate the cell cycle by directly phosphorylating target proteins or indirectly controlling protein expression (Liu et al., 2019). As an N-terminal gene of the RASSF family, RASSF9 is involved in cell growth, survival, and apoptosis. By down-regulating the expression of *p*-AKT and other related proteins, RASSF9 can restrict the AKT signaling pathway (Liu et al., 2019). In gastric cancer, the overexpression of miR-1269a can inhibit RASSF9 to activate the AKT signaling pathway, and up-regulate the transcription factors CDK2 and Cyclin D1, thereby inducing the transition of the cell cycle from the G1 phase to the S phase, promoting cell proliferation. The regulation of the PI3K/AKT signaling pathway by miR-1269a can maintain the balance between the pro-apoptotic factor Bax and the anti-apoptotic factor Bcl-2, and prevent tumor cell apoptosis (Liu et al., 2019) (Figure 1).

## The Molecular Mechanisms of miR-1269b in Tumors

### Downstream Genes of miR-1269a and Their Functions

m6A is a ubiquitous mRNA epigenetic modification in eukaryotes. METTL3 contains two domains that bind to S-adenosylmethionine (SAM) and has the activity of independently catalyzing the modification of RNA m6A. METTL3 is an important regulator of malignant tumors, which can promote the malignant biological behavior of tumor cells (Kang et al., 2021). miR-1269b is low expressed in gastric cancer, while overexpression of miR-1269b can inhibit the proliferation, migration, and invasion of tumors by targeting METTL3 (Kang et al., 2021) (Figure 1).

### The HBx/NF-κB/miR-1269b/CDC40 Axis

HBx is the smallest protein (17 kDa) encoded by the hepatitis B virus (HBV). HBx does not bind to DNA but can directly inhibit or activate transcription factors to regulate downstream genes (Kong et al., 2016). HBx can activate the transcription factor NF-κB (Yang et al., 2020). CDC40 is a splicing factor involved in cell cycle control, which can remove E-cadherin and enhance vimentin, thereby promoting tumor cell migration (Kong et al., 2016). In hepatocellular carcinoma, HBx protein can promote the introduction of NF-κB from the cytoplasm into the nucleus, thereby activating miR-1269b, up-regulating CDC40, and promoting the growth and migration of liver cancer cells (Kong et al., 2016) (Figure 2).

### miR-1269b and the PI3K/AKT Signaling Pathway

SVEP1 is one of the most important cell adhesion molecules, and it is often highly expressed in normal tissues. In liver cancer cells, down-regulation of SVEP1 expression can significantly enhance the Akt phosphorylation at Thr308, thereby promoting the proliferation and metastasis of liver cancer cells (Chen et al., 2020). miR-1269b can activate the PI3K/Akt signaling pathway by inhibiting SVEP1 in liver cancer cells, thereby promoting tumor recurrence and metastasis (Chen et al., 2020). PTEN is a known prognostic marker and tumor suppressor for non-small cell lung cancer. Its inactivation can enhance the PI3K/AKT signaling pathway, thereby promoting the development of cisplatin resistance (Yang et al., 2020) (Figure 1). miR-1269b can inhibit the PTEN/PI3K/AKT signaling pathway, thereby driving cisplatin resistance in non-small cell lung cancer.

## The Mechanism of miR-1269 Variants in Tumor

rs73239138 is a single nucleotide polymorphism located in the sequence of miR-1269a. miR-1269a rs73239138 is also associated with a reduced risk of breast cancer among women in southeastern Iran (Sarabandi et al., 2021). In gastric cancer and liver cancer cells, overexpressed miR-1269a can inhibit the apoptosis of gastric cancer cells. In contrast, the miR-1269a variant (rs73239138) can promote the apoptosis of gastric cancer cells by up-regulating the apoptotic proteins Bik, Bim, and Bak, thereby inhibiting the tumor-promoting effect of wild-type miR-1269a (Li et al., 2017). In addition, miR-1269a can inhibit the expression of tumor suppressor gene ZNF70, while miR-1269a rs73239138 can up-regulate ZNF70, thereby reducing the susceptibility to gastric cancer (Li et al., 2017). In liver cancer, miR-1269a rs73239138 can prevent miR-1269a from binding to the 3′-UTR of SOX6, thereby inhibiting the development of cancer (Xiong et al., 2015). At the same time, miR-1269a rs73239138 can disrupt the regulation of miR-1269a in the expression of NME1, SHMT1, SLC29A1, TP53, and UCK1, resulting in a poor prognosis for patients with advanced colon cancer receiving capecitabine chemotherapy (Mao et al., 2017).

However, another study of hepatocellular carcinoma showed that miR-1269a rs73239138 can promote tumor progression (Min et al., 2017). SPATS2L is ubiquitously expressed in a variety of tissues (Wang et al., 2021). SPATS2L is involved in ribosome biogenesis and translational control of oxidative stress response (Zhu et al., 2008). LRP6 is a transmembrane Wnt co-receptor necessary for the Wnt/β-catenin signaling pathway, and excessive activation of the Wnt/β-catenin signaling pathway is thought to be a key step in tumorigenesis (Dong et al., 2019). miR-1269a can down-regulate the expression of SPATS2L and LRP6, thereby inhibiting the proliferation of liver cancer cells; while miR-1269a rs73239138 can inhibit the down-regulation of SPATS2L and LRP6 by miR-1269a to promote the occurrence and development of cancer (Min et al., 2017). In the future, the role of miR-1269a rs73239138 in hepatocellular carcinoma needs further research.

**FIGURE 3:**
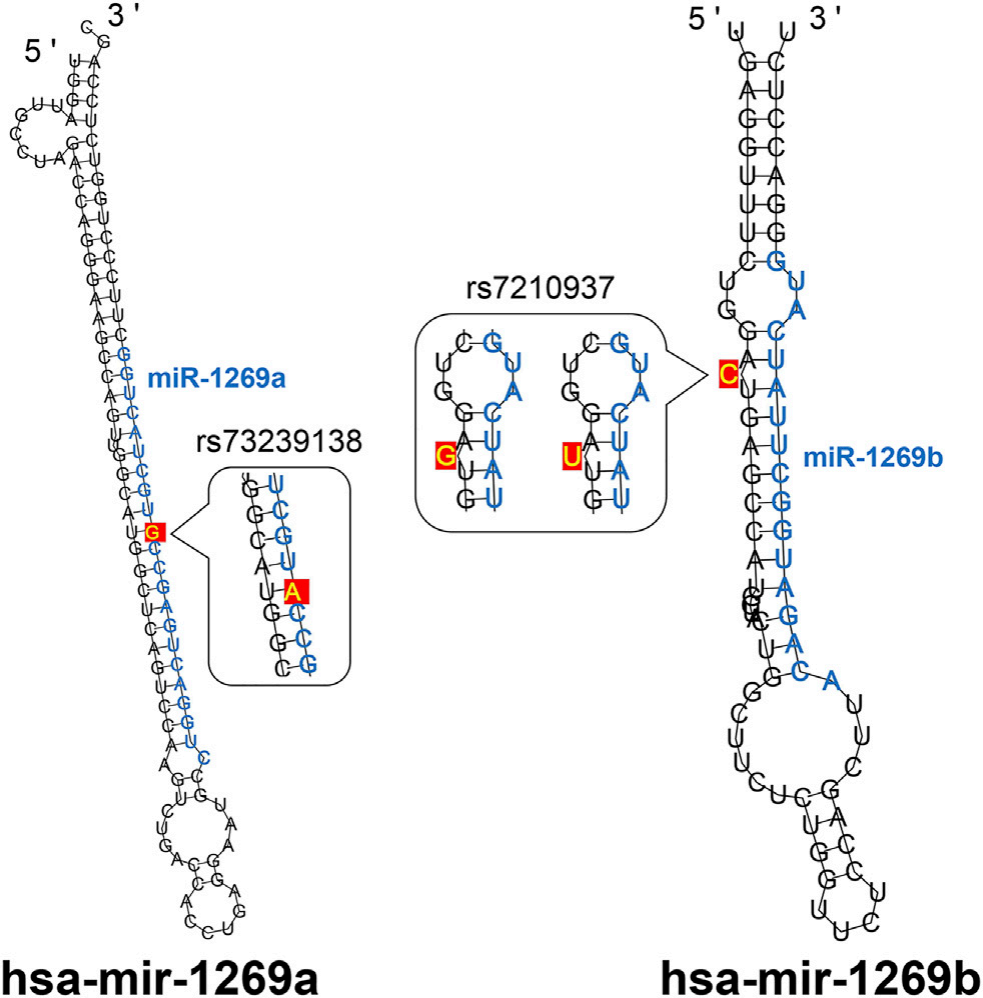
The stem-loop structures of hsa-mir-1269a and hsa-mir-1269b. The schematic diagram mainly shows the obvious differences in the stem-loop structure of hsa-mir-1269a and hsa-mir-1269b. The blue letters in the figure are the sequences of mir-1269a and mir-1269b. It is worth noting that they have the same mature sequence. In addition, the sites marked with yellow letters are known SNP site.

There is also a common genetic variant (rs7210937) of miR-1269b (Figure 3). miR-1269b rs7210937 is associated with a reduced risk of oral precancerous lesions and pharyngeal squamous cell carcinoma associated with habitual chewing of betel quid, indicating that miR-1269b rs7210937 has potential protection of cancer (Chen et al., 2016).

**FIGURE 4:**
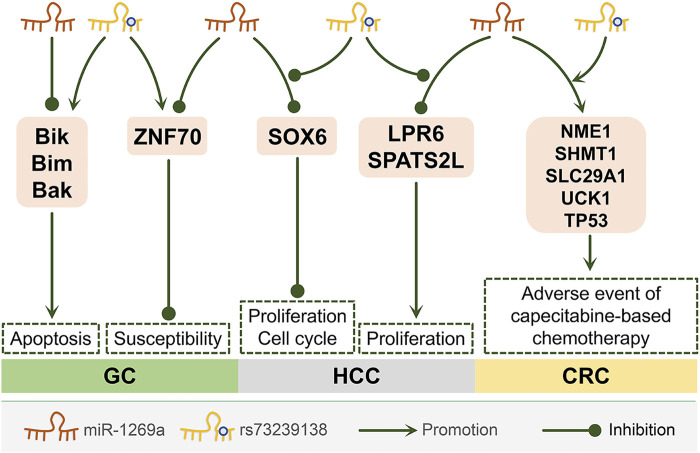
The molecular mechanism of miR-1269a rs73239138 in different cancers. miR-1269a rs73239138 can affect three cancers through five molecular mechanisms. GC, Gastric cancer; HCC, Hepatocellular carcinoma; CRC, Colorectal cancer. (Li et al., 2017; Xiong et al., 2015; Mao et al., 2017; Min et al., 2017; Wang et al., 2021; Zhu et al., 2008; Dong et al., 2019).

## Discussion

As miRNAs have been confirmed to be involved in the progression of cancer from the initial stage to metastasis, research on miRNAs has become popular (Alwani and Baj-Krzyworzeka, 2021). Numerous studies have identified miRNAs as tumor diagnostic markers and potential targets for modern cancer therapy (Alwani and Baj-Krzyworzeka, 2021). In recent years, miR-1269a and miR-1269b are involved in a variety of cancers. miR-1269a is highly expressed in 9 cancers. Growing evidence suggests that miRNAs are frequently deregulated in cancer cells, thereby affecting tumor growth, migration, invasion, apoptosis, and drug resistance (Arghiani and Shah, 2021). miR-1269a is of great significance in the diagnosis of hepatocellular carcinoma and lung cancer. In addition, the abnormal expression of miR-1269a is associated with the poor prognosis of 6 cancers. miR-1269a can regulate the occurrence and development of cancer by targeting downstream genes (CXCL9, SOX6, FOXO1, ATRX, RASSF9, SMAD7, HOXD10, and VASH1). At the same time, miR-1269a can interact with RP11-1094M14.8, LINC00261, and circASS1 in gastric cancer, lung cancer, and colon cancer, respectively. In colorectal cancer, a positive feedback loop is formed between miR-1269a and TGF-β pathway to amplify the signal of cancer metastasis, which suggests that miR-1269a is expected to become a potential therapeutic target to prevent tumor metastasis. In lung cancer and gastric cancer, miR-1269a can also promote tumor cell proliferation and cell cycle progression and inhibit tumor cell apoptosis by activating the PI3K/AKT signaling pathway and inhibiting the caspase-9-mediated apoptotic pathway, respectively.

miR-1269b is highly expressed in three types of cancer, and lowly expressed in one type of cancer. miRNA is a key regulator involved in cell carcinoma proliferation, apoptosis, invasion, metastasis, EMT, angiogenesis, drug resistance, and autophagy (Xu et al., 2018). In addition, research has shown that miRNA (such as miR-21) has an important role in promoting cell proliferation and invasion, angiogenesis, and chemical and radioresistance in non-small cell lung cancer (Cecilia et al., 2018). Our work shows that the high expression of miR-1269b is associated with the lower overall survival of patients with hepatocellular carcinoma (Ma et al., 2020) and non-small cell lung cancer (Yang et al., 2020). In non-small cell lung cancer, the high expression of miR-1269b can also promote the occurrence of cisplatin resistance (Yang et al., 2020). Accordingly, miR-1269b can affect the progression of cancer by targeting downstream genes (METTL3, CDC40, SVEP1, and PTEN). In addition, miR-1269b can also affect the progress of cancer through a series of regulatory methods, such as directly targeting the downstream gene METTL3 or by targeting SVEP1 and PTEN to drive the PI3K/AKT signaling pathway, thereby mediating tumor recurrence and metastasis. miR-1269b can also be induced by HBx to up-regulate CDC40 in an NF-κB-dependent manner to promote tumor cell growth and migration.

In hepatocellular carcinoma and gastric cancer, miR-1269a variant can reduce tumor susceptibility and inhibit tumor progression by inhibiting the effect of miR-1269a, which provides new ideas for future targeted cancer treatments. In oral cancer, the miR-1269b variant has also been proven to have potential cancer protection.

At present, most of the researches use PCR-based technology, microarray, or next-generation sequencing technology to identify miR-1269a/b. The primer sequences they used to detect miR-1269a/b are shown in Supplementary Table. They differentiated the roles of the miR-1269a and miR-1269b by detecting hsa-mir-1269a and hsa-mir-1269b. Our work shows different molecular mechanisms between miR-1269a and miR-1269b, which may be caused by the different focus of the research content. We also checked the research of miR-1269 in the NCBI GEO database and found that hsa-mir-1269a can be detected by Illumina HumanHT-12 V4.0 expression beadchip (GPL10558), however, there is no probeset to detect hsa-mir-1269b. We believe that the current paucity of miR-1269b research may be related to the lack of detection methods for hsa-mir-1269b. Therefore, more methods need to be explored in the future for the effective detection of hsa-mir-1269b to distinguish whether there is a functional difference between miR-1269a and miR-1269b.

At present, our understanding of miR-1269a and miR-1269b is still very limited, and we have not conducted a comprehensive exploration of these two oncogenic miRNAs in cancer. Studies on miR-1269a or miR-1269b are often combined with other miRNAs, and there are relatively few independent studies on miR-1269a or miR-1269b. There is still some controversy as to whether the miR-1269a variant promotes or suppresses cancer. In addition, miR-1269b has been confirmed to be highly expressed in hepatocellular carcinoma, lung cancer, and oral and pharyngeal squamous cell carcinoma. However, miR-1269b is low expressed in gastric cancer, which may be caused by some unknown regulatory mechanisms in gastric cancer, and this needs to be further studied.

In summary, miR-1269a and miR-1269b are both promising miRNAs. In the future, it is necessary to further explore the mechanism of miR-1269a and miR-1269b in a variety of cancers, to establish a richer miR-1269a and miR-1269b regulatory network. At the same time, the variants of miR-1269a and miR-1269b also have great research value, which can provide support for cancer diagnosis, targeted therapy, and prognosis prediction.

## Supplementary Material

Supplementary Table S1 The detection of miR-1269a/b in different studies.
