# Supplementary material for: miR-1269a and miR-1269b: Emerging Carcinogenic Genes of the miR-1269 Family
Source: Front Cell Dev Biol. 2022 Feb 18;10:809132. doi: 10.3389/fcell.2022.809132 (PMC8894702; doi:10.3389/fcell.2022.809132)
Supplement: Supplementary file 1 [file Table1.docx]

**Supplementary Table 1: The detection of miR-1269a/b in different studies**

| **miR-1269a/b** | **Expression pattern** | **Detection methods** | **Sequence used for detection** | **Ref.** |
| --- | --- | --- | --- | --- |
| miR-1269a |  |  |  |  |
|  | Up-regulated in HCC | Next-generation sequencing, qRT-PCR | miR-1269a primer sequence: 5'-CTGGACTGAGCCGTGCTACTGG-3' | [Cho et al., 2020](#_ENREF_10) |
|  | Up-regulated in HCC | qRT-PCR | miR-1269a primer sequence: 5'-CTGGACTGAGCCGTGCTACTGG-3' | [Gan et al., 2015](#_ENREF_14) |
|  | Up-regulated in GC | qRT-PCR | miR-1269a variant rs73239138 amplification primer sequence: 5'-ACGTTGGATGAAGTCTCATGATAGGCCATC-3', 5'-ACGTTGGATGACCTGAGGAATGCCTGGAC-3', miR-1269a variant rs73239138 extension primer sequence: 5'-CAGGGAAGCCAGTAGCA-3' | [Li et al., 2017](#_ENREF_23) |
|  | Up-regulated in GC | qRT-PCR | miR-1269a forward primer sequence: 5′- GGGCTGGACTGAGCCGTGC-3′, miR-1269a reverse primer sequence: 5′- CAGTGCGTGTCGTGGAGT-3′ | [Zhang et al., 2020a](#_ENREF_50) |
|  | Up-regulated in GC | qRT-PCR | miR-1269a reverse-transcription primer sequence: 5′-GTCGTATCCAGTGCGTGTCGTGGAGTCGGCAATTGCACTGGATACGACTCAGGTC-3′, miR-1269a forward primer sequence: 5′-ATCCAGTGCGTGTCGTG-3′, miR-1269a reverse primer sequence: 5′-TGCTGGTCATCGTGCCGAG-3′ | [Liu et al., 2019](#_ENREF_24) |
|  | Up-regulated in NSCLC | RT-PCR | miR-1269a primer sequence: 5'-GACTGAGCCGTGCTACTGGAAA-3' | [Wang et al., 2020](#_ENREF_40) |
|  | Up-regulated in NSCLC | qRT-PCR | miR-1269a forward primer sequence: 5′-ACGTTGGATGAAGTCTCATGATAGGCCATC-3′, miR-1269a reverse primer sequence: 5′-ATTGCGTGTCGTGGAGTCGGCAATGC-3′ | [Jin et al., 2018](#_ENREF_17) |
|  | Up-regulated in LC | qRT-PCR | miR-1269a reverse-transcription primer sequence: 5′-GTCGTATCCAGTGCGTGTCGTGGAGTCGGCAATTGCACTGGATA-CGACCCAGTAGC-3′, miR-1269a forward primer sequence: 5′-GACTGAGCCGTGCTACTGG-3′, miR-1269a reverse primer sequence: 5′-TGTCGTGGAGTCGGCAATTG-3′ | [Guo et al., 2020](#_ENREF_15) |
|  | Up-regulated in CRC | qRT-PCR | miR-1269a forward primer sequence: 5′-GCTGGACTGAGCCGTGC-3′, miR-1269a reverse primer sequence: 5′-CAGTGCGTGTCGTGGAGT-3′ | [Xiong et al., 2021](#_ENREF_44) |
|  | Up-regulated in Glioma | qRT-PCR | miR-1269a reverse-transcription primer sequence: 5′-GTCGTATCCAGTGCGTGTCGTGGAGTCGGCAATTGCACTGGATACGACCCAGTA-3′, miR-1269a forward primer sequence: 5′-CTGGACTGAGCCGTGC-3′, miR-1269a reverse primer sequence: 5′-CAGTGCGTGTCGTGGA-3′ | [Zhang et al., 2020b](#_ENREF_52) |
|  | Up-regulated in AML | Transcriptome sequencing, qRT-PCR | Not provided | [Li and Ge, 2021](#_ENREF_22) |
|  | Up-regulated in HCC | Next-generation sequencing | Not provided | [Wojcicka et al., 2014](#_ENREF_41) |
|  | Up-regulated in HCC, PC, ESCC, CRC and NSCLC | qRT-PCR | Not provided | [Yang et al., 2014](#_ENREF_47); [Bu et al., 2015](#_ENREF_6); [Scaravilli et al., 2015](#_ENREF_36); [Min et al., 2017](#_ENREF_29); [Bai et al., 2021](#_ENREF_4); [Le and Le, 2021](#_ENREF_21) |
|  | Up-regulated in HCC and LC | RT-PCR | Not provided | [Xiong et al., 2015](#_ENREF_43); [Elemeery et al., 2017](#_ENREF_13); [Bao et al., 2018](#_ENREF_5) |
| miR-1269b |  |  |  |  |
|  | Up-regulated in HCC | qRT-PCR | miR-1269b reverse-transcription primer sequence: 5′-GTCGTATCCAGTGCAGGGTCCGAGGTGCACTGGATACGACCCAGTAGC-3′, miR-1269b forward primer sequence: 5′-TGCGCTGGACTGAGCCATGC-3′, pre-miR-1269b-qPCR-S primer sequence: 5′-CAGGCTGGGAGAAAGACC-3′, pre-miR-1269b-qPCR-AS primer sequence: 5′-GGAAGTTGGCTCACATAATC-3′, pre-miR-1269a-qPCR-S primer sequence: 5′-TGGATTGCCTAGACCAGGG-3′, pre-miR-1269a-qPCR-AS primer sequence: 5′-GCTGGAGACCAGGGAAGCCAG-3′ | [Kong et al., 2016](#_ENREF_20) |
|  | Up-regulated in HCC | Next-generation sequencing, qRT-PCR | miR-1269b primer sequence: 5'-CUGGACUGAGCCAUGCUACUGG-3' | [Chen et al., 2020](#_ENREF_8) |
|  | Down-regulated in GC | qRT-PCR | miR-1269b reverse-transcription primer sequence: 5′-GTCGTATCCAGTGCAGGGTCCGAGGTGCACTGGATACGACCCAGTAGC-3′, miR-1269b forward primer sequence: 5′-TGCGCTGGACTGAGCCATGC-3′ | [Kang et al., 2021](#_ENREF_18) |
|  | Up-regulated in NSCLC | qRT-PCR | Not provided | [Yang et al., 2020](#_ENREF_46) |
|  | Up-regulated in OPSCC | RT-PCR | Not provided | [Chen et al., 2016](#_ENREF_7) |

HCC, Hepatocellular carcinoma; GC, Gastric cancer; NSCLC, Non-small cell lung cancer; LC, Lung cancer; CRC, Colorectal cancer; ESCC, Esophageal squamous cell carcinoma; PC, Prostate cancer; AML, Acute myeloid leukemia; OPSCC, Oropharyngeal squamous cell carcinoma; qRT-PCR, quantificational real-time polymerase chain reaction; RT-PCR, reverse transcription-polymerase chain reaction
